# *TechMaps*: exploring technology relationships through patent information based proximity

**DOI:** 10.3389/frma.2023.1096226

**Published:** 2023-07-05

**Authors:** Eduardo Perez-Molina, Fernando Loizides

**Affiliations:** ^1^ETSIT, Universidad Politécnica de Madrid, Madrid, Spain; ^2^School of Computer Science and Informatics, Cardiff University, Cardiff, United Kingdom

**Keywords:** patent analytics, technology maps, technology visualization, patent databases, patent classification

## Abstract

Our work provides a novel method for rich information discovery about the evolution of technical fields and company developments through patent relationships. A new exploratory method and graphical tool to discover technology proximity based on patent classification information are introduced. By technology we mean a technical field (defined by an International Patent Classification—IPC—code or a combination of them) or an organization (such as a tech company, research center, or institution). A single data structure is used for characterizing both technical fields and organizations, to visualize them as items of the very same body. This new method generates two graphs: the first graph, the *TechnologyMap*, visualizes technology items in a 2D plot wherein technical fields and companies will appear positioned relative to each other; the. A second graph, the *Focused TechnologyMap*, visualizes technology items with respect to a selected one, the *focus*, which is located in the center of a circle whose radii correspond to the complete set of IPC codes. This article represents the process and algorithms used for production of the graphs, and solidifies the assumptions of validity by presenting two of the many successful test cases to which it was applied.

## 1. Introduction

Discovering the proximity between technical fields have been proved is important for the global understanding of science and technology, facilitating, for example, knowledge technology transfer, research collaboration between institutions (Woerter, 2012), or the identification of technology opportunities (Jaffe, 1986). By discovering technology proximity, we mean to assess the similarity of technologies, namely the share of knowledge and techniques. In the case of characterizing technology using patent information, proximity could be evaluated by identifying the commonalities at classification level (Jaffe, 1986; Simon and Sick, 2016; Alstott et al., 2017). In other words, two technologies are closer to each other than a third one when the first two have more classification information in common in any way that the third one (Boyack et al., 2000; Schoen et al., 2012; Woerter, 2012; Okubo, 2017).

Revealing the technical fields where companies have industrial or research developments could play a key role in the decision-making of technology players in areas such as analysis of competition (Schoen et al., 2012), firm collaboration (Simon and Sick, 2016), or mergers and acquisitions (Simon and Sick, 2016).

Our objective with the present work is the visualization of the relative position of technology, that is placing in a graph technical fields and tech companies with respect to each other. The idea is to use a single data structure based on patent classification information for characterizing both, technical fields and organizations (company, research institution, or university lab), in order to visualize them as items of the very same body, namely technology. In this way, observations such as proximity or dynamics could be extended from technical fields and tech companies to each other.

We have characterized a technical field or an organization (company, research institution, or university group) by a single data structure, the *TechSpectrum*, formed by the aggregation into bins^1^ of the IPC codes assigned to a set of patents and its prior art, mixed in a specific proportion, and ordered according to the IPC (Perez-Molina, 2018).

**Figure 1:**
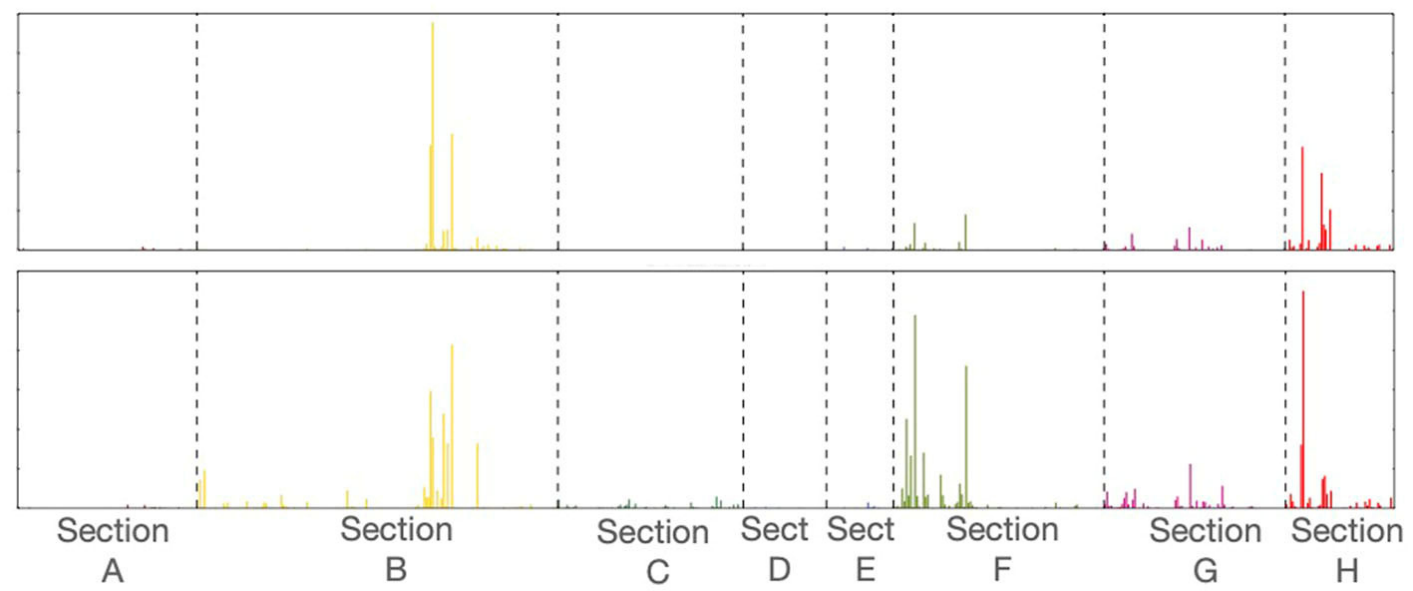
*TechSpectrum* for the IPC code *B60L* **(top)** and *Toyota Motors* **(bottom)** at IPC–*subClass* level. A different color is assigned to each IPC-*Section* in order to facilitate the interpretation of the graph.

This binning and ordering is done at different IPC granularities, generating a particular *TechSpectrum* for each IPC level^2^ (see for example in Figure 1 the *TechSpectrum* graph at IPC-SubClass level for propulsion of electric vehicles—IPC code *B60L*—and *Toyota Motors*).

A *TechSpectrum* is a histogram, each bin of which corresponds to an specific IPC code, and it could be considered as an ax of a multidimensional space, namely the IPC space. The bin values would be in consequence coordinate values. Under this perspective, the visualization of technology, that is a technical field or a tech companies represented by selected sets of patents and its prior art citations, would be merely be the representation of dots in the positions corresponding to the respective set of coordinates values, namely the bin values.

We have developed an analysis tool which generates two graphs, the *Technology Map*—*TechMap*—and the *Focused Technology Map*—*F-TechMap*—.

The first graph, the *TechMap*, visualizes a given set of items, thus technical fields and tech companies, in a 2D plot in such a way that those related items they will appear closer between them that the unrelated ones.

The fact that our system has a high dimensionality, (the dimension is 8, 132, 651, and 7,590 at IPC–*Section, Class, subClass*, and *Group*, respectively)^3^, makes difficult a straightforward representation difficult. An approach to visualize such high dimensional spaces is, first to reduce the dimensionality to two dimensions, and then generate a visualization in a 2D plane. For this task we have applied *MultiDimensional Scaling*—*MDS*—, the downside of such an appreciable reduction of dimensionality is the introduction of a geometric distortion (Colange et al., 2019).

Note that for our aim of exploring proximity between technologies, *MDS* has the interesting feature of reducing the dimensionality to two dimensions preserving as much as possible the relative distances between the items to be visualized. In other words, items that are close in the *IPC* space stay close to each other, and those far stay far. On the other hand, the absolute positioning of the items within this visualization is meaningless, namely the fact that a given item is located in a particular position in the 2D plane provides no information, only the closeness or farness to any other item is meaningful (Borg and Groenen, 2005).

*MDS* visualizes the items in a 2D plane based on the distance information using a nonlinear dimensionality reduction, so the distances between the elements are is the key information to gather. This information is technically provided as a distance of dissimilarity matrix.

The distance between items to visualize is computed with the *soft cosine* (Sidorov et al., 2014) algorithm, wherein the features are the IPC codes, and the similarity measure between features is computed by applying a hierarchical similarity measure for hierarchical classification schemes (Caspersen et al., 2017).

Our second graph, the *Focused TechMap*—*F-TechMap*—is inspired on by the work of Urpa and Anders (2019), and takes the shape of a circular—radial graph with the focus at the center of the circle and a set of radii, each corresponding to a single IPC code at a certain level of resolution. Once an item between the technical fields or companies present in the *TechMap* is chosen as to be the focus, then every other item to be visualized is located at the computed distance on a *radius* corresponding to a given IPC-code.

The *F-TechMap* also uses the distances computed with the *soft cosine* algorithm, and it is a complementary graph to the *TechMaps* because it immediately uses computed distances avoiding the distance distortions produced by MDS in its projection on the 2D plane. The dynamic version of these two graphs will provide rich information about the evolution of companies' technical developments and the evolution of technical fields.

Two study cases are presented with some technical fields and tech companies. The data collection of the study cases is done using *PatStat–online*^4^, an EPO's database in the field of patent intelligence and statistics^5^. A set of queries is executed in PatStat to collect the patents and citations of the technological fields or companies under study. In all our study cases, patents are limited to the 1980–2015 range of years in order to have a large interval of time^6^. The patent documents collected are also constrained to be published by the USPTO^7^ in order to avoid duplication.

The research question of the present work is what can we learn about tech companies' development activities, and about technical fields' evolution by visualizing patent classification information-based maps.

The potential contribution of our work in the field of innovation management lies in the creation of a new graphical tool for the relative positioning of technologies (technical fields or tech companies) based on patent classification information, improving the perception and analysis of research and industrial activities of technology players. In the field of history of technology, our procedure contributes by facilitating the study of the evolution of specific technical fields and tech companies.

The rest of the paper is organized as follows: Section 2 discloses related works. Section 3 discloses the procedure to generate our graphical tool. Section 4 presents some case studies. Section 5 presents a discussion and the applications of our tool. Section 6 outlines future research and conclusions.

### 1.1. Related works

The visualization of technology has long been used for long as a tool for analyzing technology and innovation (Geisler, 1962). The range of visualizations goes from relatively simple graphs to visually rich and complex rendering such as Yoon and Magee (2018) for exploring technology opportunities and of Boyack et al. (2002) to visualize landscapes.

Patents are a fundamental component of the technology ecosystem, and patent information is employed extensively for technology visualization. For example, Yoon and Park (2004) have disclosed an analytical tool for high-technology trends which visualizes patent networks based on text mining; or Liu and Zhu (2009) presented a system to visualize patent citations using web mining. vonWartburg et al. (2005) have studied multistage patent citations to assess inventive progress. Moreover, maps of science or technology have been also produced by some authors such as Yan and Luo (2017) for measuring patent distances, Leydesdorff et al. (2013) using *Web-of-Science* categories, or Boyack et al. (2000) using patent citation networks for positioning specific patents on the landscapes.

Evaluating technological proximity based on patent information has been investigated by numerous authors. Yan and Luo (2017) presented an overview of distance measurement for patent mapping. Simon and Sick (2016) disclosed the computation of technology distance based on patent classification codes. And, Schoen et al. (2012) studied the evaluation of technological proximity based on patent information, specifically using co-classification.

Considering the above-mentioned *TechSpectrum* merely as a histogram makes it possible to compute technological distances by computing histogram distances. Such methods are disclosed between other by Werman et al. (1985), Serratosa and Sanfeliu (2006), Strelkov (2008), or Ma et al. (2010). However, in our case the histograms are very heterogeneous, and especially some IPC *classes* have unimodal histograms in comparison with big tech corporations which have highly multimodal distributions. Additionally, the absolute figures of each frequency bin are also extremely different for some technologies represented by specific IPC codes and tech companies. These disparities in the histograms present problems resulting from the histogram normalization necessary for computing the distance based on histogram shape.

Considering the *TSpectrum* as a vector of the IPC space, the first solution is to use the *Euclidean* distance of the two vectors, that is the two *TSpectrum*s. However the resulting distance is very sensitive to strong differences among the coordinate values of both vectors (Simon and Sick, 2016).

An alternative is to compute the *Cosine* distance (Sidorov et al., 2014) because the result does not depend on the magnitudes of the vectors, but only on their angle. It remains the problem that not every *IPC* code is equidistant—orthogonal—because a given bin (an IPC code at a certain level) is usually much closer to other codes that belong to the same IPC *section, class*, or *sub-class* than to those that belong to another^8^. An improvement to this issue is the *Soft cosine* distance (Sidorov et al., 2014), which includes a factor among the different coordinates to take into account a certain *proximity* or *similarity* among them, meaning in our case a similarity factor between IPC codes.

Similarity in hierarchical structures, such as a classification tree, conceptual taxonomy, or ontology, can be computed by using only the structural information of the tree, such as the concept similarity computation presented by Wu and Palmer (1994), which uses the number of nodes related to the concerned concepts. Li et al. (2003) discloses the computation of semantic similarity based on structural semantic information from a lexical taxonomy as a function of path length and depth between words. Caspersen et al. (2017) have disclosed a measure of similarity between labels in a hierarchical classification scheme for automatic classification.

The visualization of multidimensional data in a 2D plane was studied by numerous authors (Torgerson, 1952; Young, 1987; Tenenbaum et al., 2000; Borg and Groenen, 2005; Lespinats et al., 2007). The basis of *MultiDimensional Scaling*—*MDS*—were was disclosed by Torgerson in his seminal work of 1952 (Torgerson, 1952). Young (1987) and Borg and Groenen (2005) exposed some applications. An interesting use of *MDS* for exploring high-dimensional data is presented by Urpa and Anders (2019).

## 2. Method: technology maps generation procedure

In our work a technology is defined as a set of patent documents assigned to a technical field or belonging to a tech company. The set of patents owned to by an inventor, or a group of inventors could also be considered, but this work is limited to tech fields and companies.

The patents are collected from *PatStat*, an EPO's database in the field of patent intelligence and statistics. A set of *SQL*–queries is executed in Patstat to collect the selected patents—and its prior art citations—of the technological fields or companies under study. The patent documents collected are constrained to first, to the range of years, from 1980 to 2015, in order to have a large interval of time, and second, to be published by the USPTO to avoid duplication^9^.

The *TechMap* generation procedure is outlined in the following five steps:

**Step 1**: Data gathering.—First, we collect the set of patents published for the specific technical fields or tech companies to be visualized, then its cited prior art is also collected and mixed, forming a final collection. In this final collection, the prior art is weighted to a certain percentage which experimentally we have set to 10%;, the idea of this reduction is to keep the basic composition of the initial set of patents but enriching it with the prior art citations.

**Step 2**: *TechSpectrum* generation.—For each final collection, the classification IPC codes are identified, and ordered in bins according to the IPC at the first four levels of classification resolution^10^.

**Step 3**: Distance computation.—A matrix of distances between every and each and every item (technical fields or tech companies) to be visualized in the Tech Maps is computed using the *soft cosine* distance (Sidorov et al., 2014), which is uses the following formula:


(1) $$\begin{array}{ll} SoftCosDist_{AB}=\frac{\sum_{i}^{N}\sum_{j}^{N}\left(S_{ij}A_{i}*B_{j}\right)}{(\sqrt{(\sum_{i}^{N}\sum_{j}^{N}S_{i_{j}}(A_{i}*A_{j}))}*(\sqrt{(\sum_{i}^{N}\sum_{i}^{N}S_{ij}(B_{i}*B_{j}))}} & \;\;\;\;(1) \end{array}$$

*A* and *B* represents vectors formed by bins of the final collections of two specific technologies (tech fields or companies). *A*_i_ and *B*_j_—are the bin i and j of each vector, namely the IPC codes ordered according to the IPC at position *i*, and *j* of the two specific technologies.

For computing the *similarity* factor among each possible couple of IPC codes—S_i__j_—we have adapted the method proposed by Wu and Palmer (1994) to structural information of the hierarchical IPC scheme, namely the number of IPC levels between the path of each couple of IPC-codes *C*_i_ and *C*_j_ resulting in the following formula:


(2) $$\begin{array}{l} Similarity\left(C_{i},C_{j}\right)=\frac{N_{I_{J}}}{N_{i}+N_{j}+\left(2*N_{I_{J}}\right)} \end{array}$$

*N*_i_, *N*_j_, and *N*_IJ_ are the number of nodes on the path from *C*_i_ to *C*_IJ_, from *C*_j_ to *C*_ij_, and from *C*_ij_ to the *Root* node, where *C*_IJ_ is the least common IPC code of *C*_i_ and *C*_j_.

In this manner, four distance matrices are formed with the *soft cosine* distances between every possible couple of items corresponding to the first four IPC levels. Then, we mix these matrices to form a *global* matrix^11^.


(3) $$\begin{array}{l} GlobalDistance_{i_{j}}=K_{\text{S}}*d_{{\text{S}}_{ij}}+K_{\text{C}}*d_{{\text{C}}_{ij}}+K_{\text{SC}}*d_{{\text{SC}}_{ij}}+K_{\text{G}}*d_{{\text{G}}_{ij}} \end{array}$$

K_S_, K_C_, K_SC_, and K_G_, and d_C_, d_S_, d_SC_, and d_G_ are the mix factors and the *soft cosine* distances at IPC *Section, Class, SubClass*, and *Group*, respectively. We have experimentally set the parameters to K_S_=1, K_C_=2, K_SC_=3, and K_G_=5.

**Step 4**: *MDS* computation.—The dimensionality reduction is made at the *global* level by *MDS* using the *SKlearn* python library^12^. The *MDS* results in a collection of 2D coordinates corresponding to each item to be visualized.

**Figure 2:**
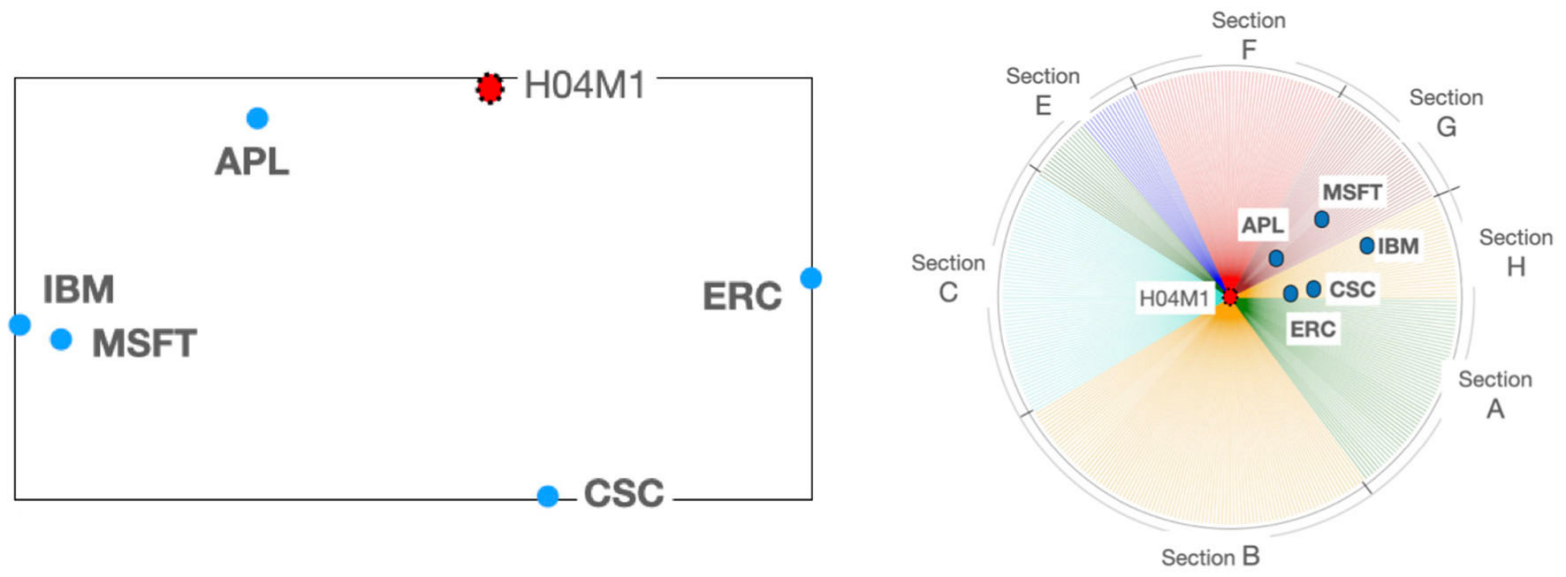
*TechMap* **(left)** with a technical field: telephone set—*H04M1*—and some computer and telecom companies: *Apple, Cisco, Ericsson, IBM*, and *Microsoft*, and the corresponding *F-TechMap* **(right)** with focus at *H04M1*.

**Step 5**: *TechMap* visualization.—The *MDS* results are visualized using the *MatPlotLib* python library (Devert, 2014). In the graphs, in order to facilitate the understanding, the tech companies are visualized as blue dots and the technical fields as red dots. An example of a *TechMap* with an IPC code^13^ and some tech companies is shown in Figure 2, left graph.

The *Focused TechMap* generation procedure is outlined in the following three steps:

**Step 1**: *TechMap* generation.—The global *TechMap* is generated and visualized for a given set of items (technical fields or tech companies) executing the *TechMap* generation procedure explained above.

**Step 2**: *Focus* selection.—Once the *TechMap* is generated, we select one of the visualized items to be the *focus* of the graph.

**Step 3**: *Focused TechMap* visualization.—The focus is drawn at to the center of a circle wherein a set of radii is in turn plotted; this set contains a radius corresponding to each IPC code. Then, each item is drawn at its real computed distance to the focused item, on the radius corresponding to its main IPC code. The main IPC code for technical fields and tech companies is the IPC code with the highest figures. An example of a *focused TechMap* with an IPC code and some tech companies is shown in Figure 2, right graph.

## 3. Results: study cases

As mentioned above, the data collection of the study cases is done using *SQL* queries in *PatStat* to gather the selected patents, and its their prior art citations, and the corresponding assigned IPC codes. The dimensionality reduction, and visualization is implemented with MDS, and the distance or dissimilarity matrix in all the study cases is computed using the *SoftCosine* distance.

For the study cases, we have selected two technical fields, namely: medical science—*A61*—and vehicle technology—*B60*—, and the following companies^14^: *Apple* (APL), *Boston Scientific* (BSc), *Cisco* (CSC), *Ericsson* (ERS), *Ford Motor* (FRD), *GE-medical* (GEm), *General Motors* (GMt), *Hyundai Motors* (Hyu), *IBM* (IBM), *Medtronic* (MDT), *Nissan* (Nss), *Microsoft* (MSFT), *Nihon Kohden* (NKH), *Olympus-medical* (OLYM), *Omron-medical* (OMR), *Philips* (PHLP), *Shimadzu* (SHI), *Siemens Healthcare* (SIEM), *Toyota Motors* (TYT), and *Volkswagen* (VW).

We have chosen the medical field for personal and academic interest since the origin of this work is in a research project in the biomedical engineering field. The second study case, vehicle technology, was chosen for its general interest as a technology in undergoing important transformation at present and because it encompasses multidisciplinary properties. On the other hand, the set of tech firms was selected to have a heterogeneous group with big- and mid-size corporations from different sectors and geographic origins.

### 3.1. Medical technologies

In this study case we will use our tool to study some tech companies related with to medical technologies.

**Figure 3:**
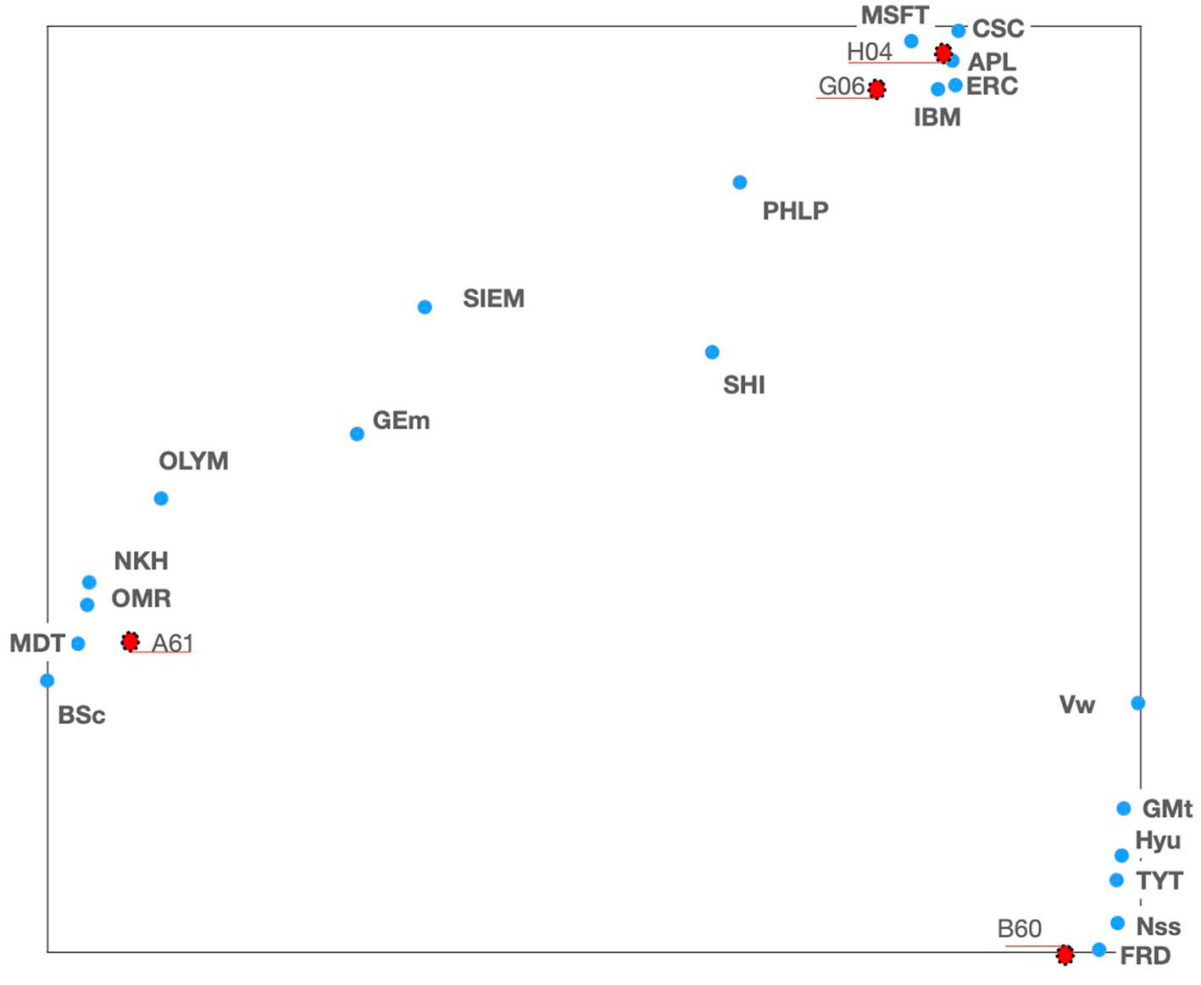
*TechMap* for some technical fields corresponding to IPC *classes* (red dots), and some tech companies (blue dots).

Let us start by exploring the location of our complete set of tech companies and the major technical fields where they are supposed to develop its their activities. Therefore, we will generate a *TechMap* with all the companies of our set and four technical fields: medical science—*A61*—, vehicle technology—*B60*—, computation—*G06*—, and telecommunications—*H04*—. Figure 3 shows this *TechMap*, wherein the red dots represent the IPC code and blue dots represent the tech companies.

Looking at Figure 3, we note three groups of companies: A first group around medical science, IPC code *A61*, and formed by the companies *Boston Scientific, GE-medical, Medtronic, Nihon Kohden, Olympus-medical, Omron-medical*, and *Siemens Healthcare* (see mid-left area in Figure 3). A second group close to computer science and telecommunication, IPC codes *G06* and *H04*, and formed by *Apple, Cisco, Ericsson, IBM*, and *Microsoft* (see top-right area in Figure 3); and a third group close to vehicle technologies, IPC code *B60*, formed by the automotive companies *Ford Motors, GM, Hyundai, Nissan, Toyota*, and *Volkswagen* (see bottom-right area in Figure 3). Finally, *Philips* and *Shimadzu* are located somewhere in between the “medical” and the “computer-telecom” groups.

**Figure 4:**
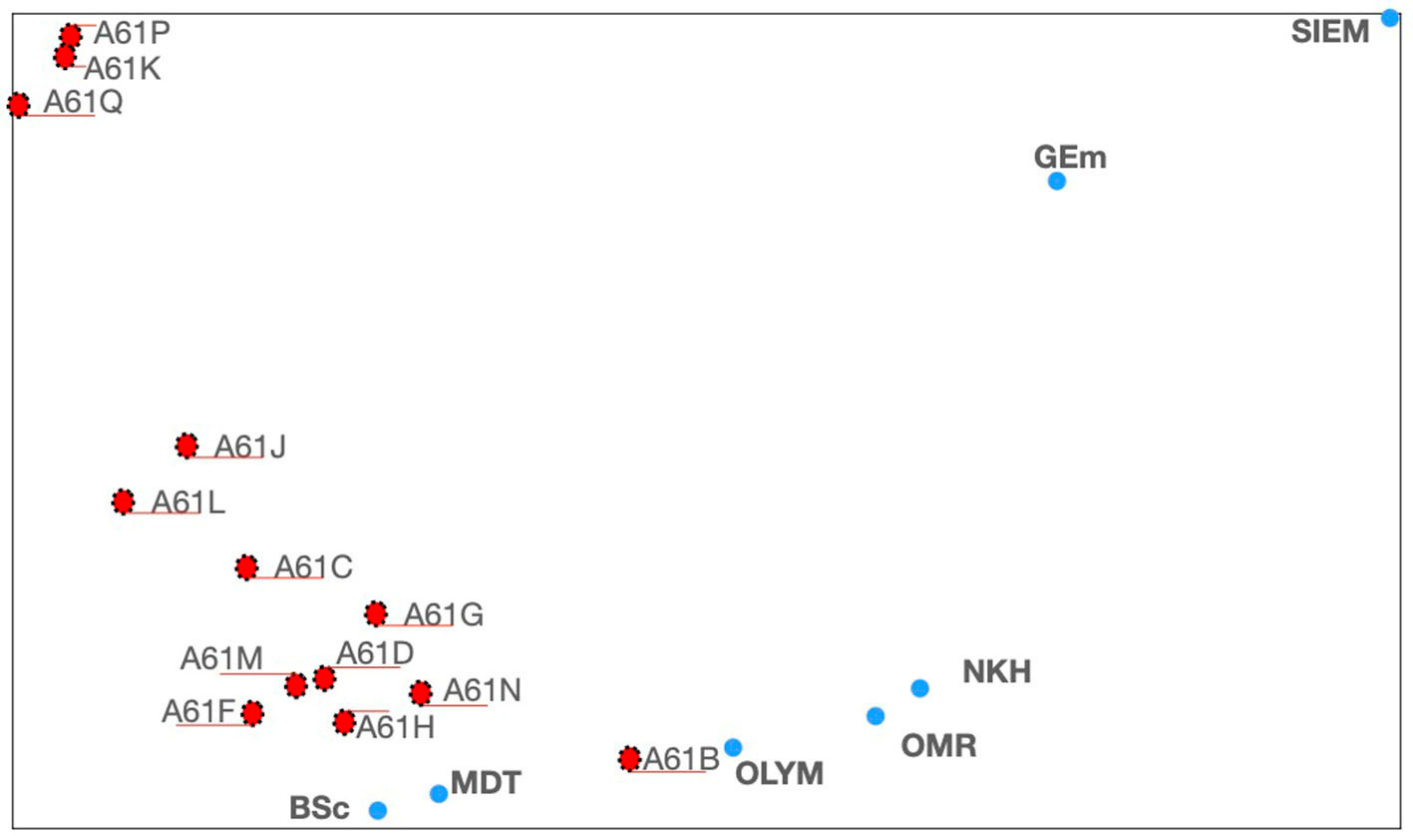
*TechMap* for medical companies and—*A61*—IPC *subclasses*. Appendix A contains the company names and its the abbreviations used in this figure.

Now, we will have a deeper look into the medical area going down a level from *A61*—IPC *class*—by generating a *TechMap* containing all the *A61* IPC *subclasses* and the companies around the medical area, *Boston Scientific, GE-medical, Medtronic, Nihon Kohden, Olympus-medical, and Omron-medical* and *Siemens Healthcare* (see Figure 4).

It is interesting to highlight that by going deeper in the IPC scheme by replacing an IPC *class* by its subclasses, or in a more general way by replacing an IPC code at a certain level by its set of IPC sub-levels, we are doing a sort of conceptual zoom rather than a zoom-in graphical operation, meaning that each item in the map is recomputed and located accordingly.

The new generated *TechMap* shows in Figure 4 that the companies are grouped in a first set with *Boston Scientific, Medtronic, Olympus-medical, Nihon Kohden*, and *Omron-medical*, and a second set with *Siemens Healthcare* and *GE medical* (see Figure 4 at the bottom and the top-right, respectively). The first group is located around diagnosis techniques—*A61B*—. It appears from Figure 4 that *Nihon Kohden, Omron-medical*, and *Olympus-medical* are particularly close to this technique, whereas *Boston Scientific* and *Medtronic* are in turn located close to *A61F, A61H*, and *A61N*.

**Figure 5:**
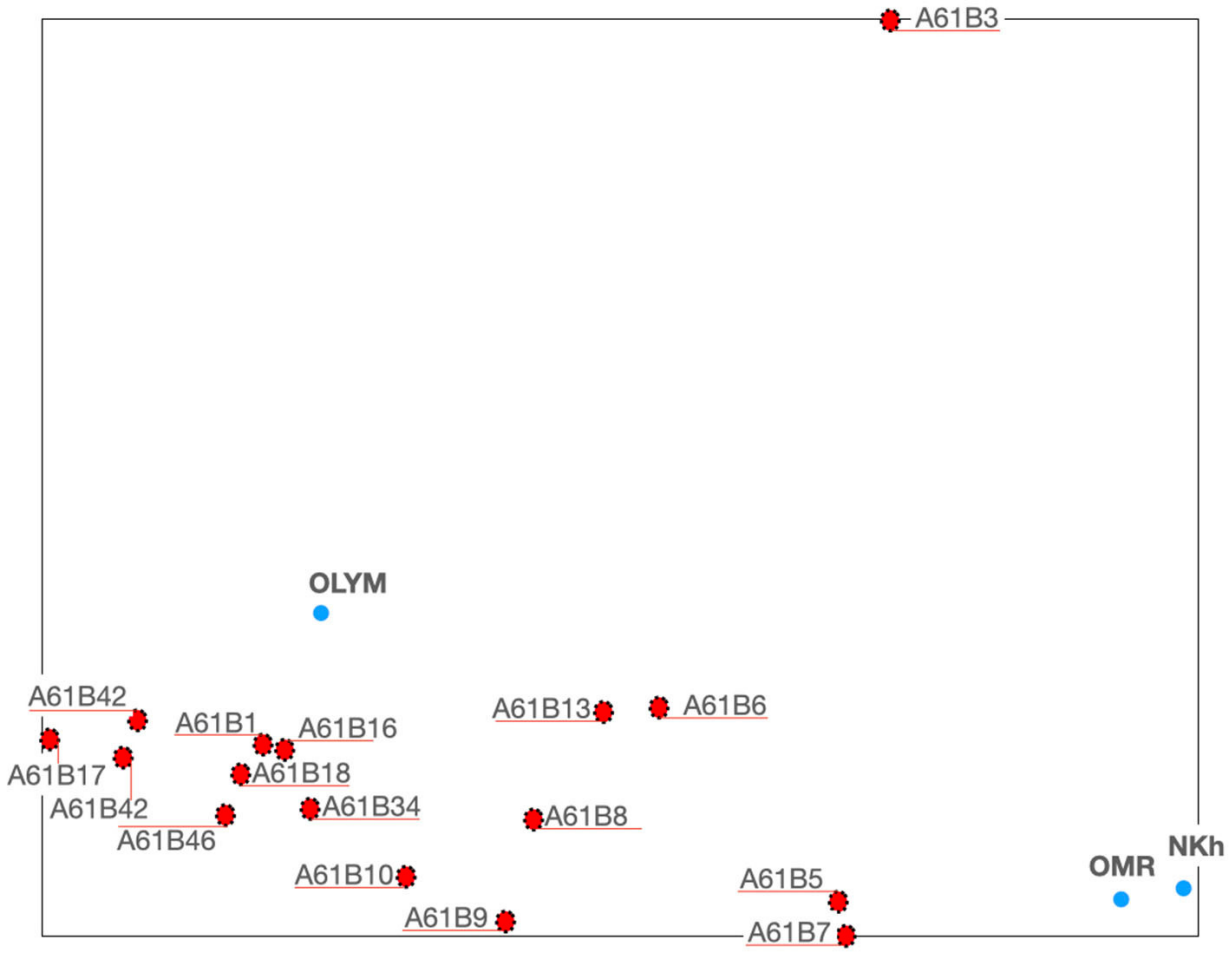
*TechMap* with all *A61B* groups and *Nihon Kohden, Omron-medical*, and *Olympus-medical*.

Let us go into more detail around *Nihon Kohden, Omron*, and *Olympus* and *A61B*. This is done by going one IPC level down from *A61B*, so thereby generating a new *TechMap* with the IPC *A61B group*s^15^ and the three tech companies (see Figure 5).

Two observations can be made from this *TechMap*: firstly, *Nihon Kohden* and *Omron-medical* are grouped and close to *A61B5* and *A61B7*, whereas *Olympus-medical* is close to a group of IPC *groups* formed by *A61B1, A61B16*, and *A61B18*. Secondly, some IPC *groups* appear in clusters, one with *A61B5* and *A61B7*, and another cluster with *A61B1, A61B18*, and *A61B16*.

**Figure 6:**
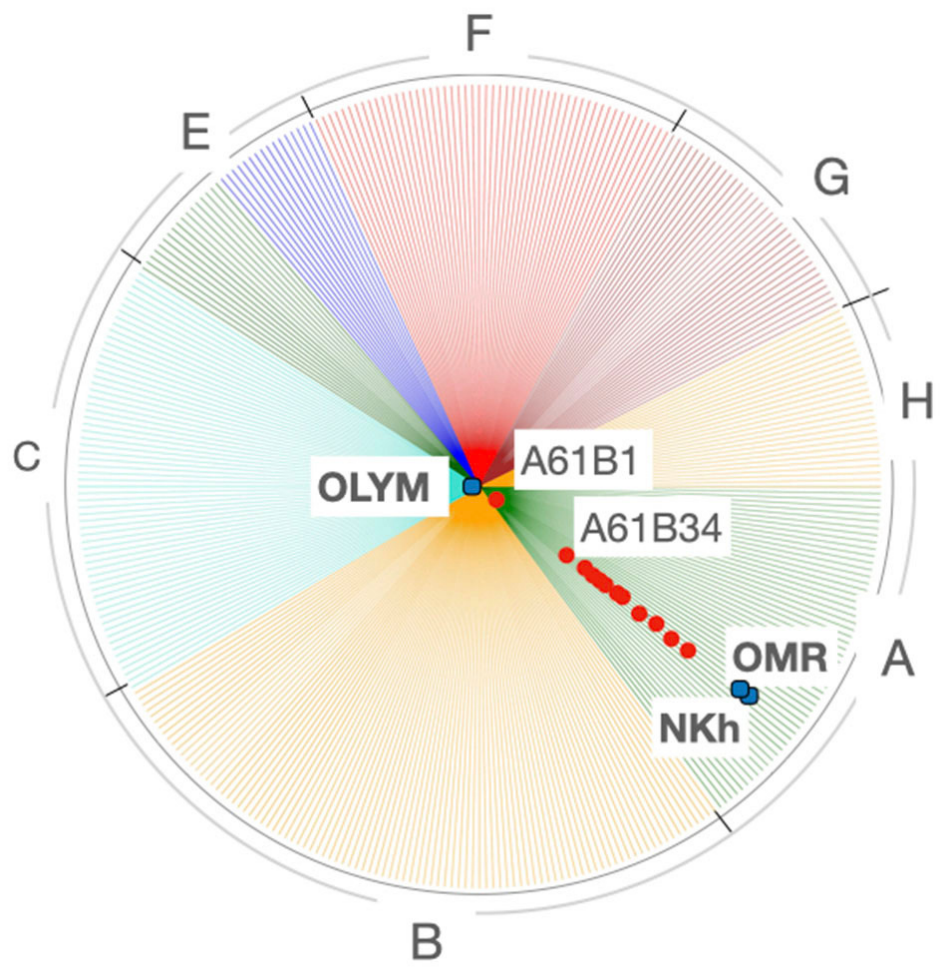
*Focused TechMap*s with focus on *Olympus-medical*.

*Olympus-medical* appears in Figure 5 close to a certain number of technical fields, so we will now use the *focused TechMap* tool to perceive without any distance distortion the closest, and therefore the most important, technical fields for that company. The *focused TechMap*s with *focus* on *Olympus-medical* shows that endoscopes—*A61B1*—are by far the main technical field of its developments followed by computer-aided surgery—*A61B34*—(see Figure 6).

These maps, and the positioning of companies wherein, are consistent with real data—the patent documents—owned by these companies. For example, in Figure 3, *GE-medical* and *Siemens Healthcare* appear on the periphery of the *A61* cluster, toward *G06*. The analysis of the patent portfolio of these two companies reveals that their patents are mainly classified in *A61, G06*, and *G01*^16^, in on the other hand, the patent portfolio of *Boston Scientific, Medtronic, Nihon Kohden, Olympus-medical*, and *Omron-medical* are virtually only assigned to *A61*. In Figure 4, *GE-medical* and *Siemens Healthcare* continue to be uncoupled from the rest of the medical companies. Additionally, this Figure shows a cluster of three medical technologies: *A61K*^17^, *A61P*^18^, and *A61Q*^19^ clearly separated from the rest of the medical technologies. Note that these three technologies (*A61K, A61P*, and *A61Q*) are dealing with medical or toiletry preparations, and they are the only medical technologies at this IPC level that are highly co-classified in IPC-codes within—*Section C*—chemistry—. Furthermore, Figure 5 shows the grouping of *Omron-medical* and *Nihon Kohden* far from Olympus medical;, this grouping is consistent with the fact that their patent portfolios are mostly in technologies covered by the IPC *Group* A61B5^20^, whereas *Olympus-medical* portfolio contains patents classified mainly in the IPC *Group* A61B1^21^ and in very low figures in A61B5.

### 3.2. Automotive technologies: electric vehicles

In this study case, we will study the evolution of the companies which are located in the *TechMap* of Figure 3 close to vehicle technology, namely *Ford, General Motors, Hyundai Motors, Nissan, Toyota Motors*, and *Volkswagen* (see at the bottom right of Figure 3). We will study these companies in relation to electric vehicle technology. Moreover, in this case the data will be visualized in time-lapses of 5 years (from 1985 to 2015) to have a dynamic perception.

Electric vehicle technology is covered by the IPC subclass *B60L*. So, we will generate a first *TechMap* containing this subclass and our set of motor car companies for the whole time interval (from 1985 to 2015) to have an overview of the relative positioning of the companies to the Electric vehicles technology.

**Figure 7:**
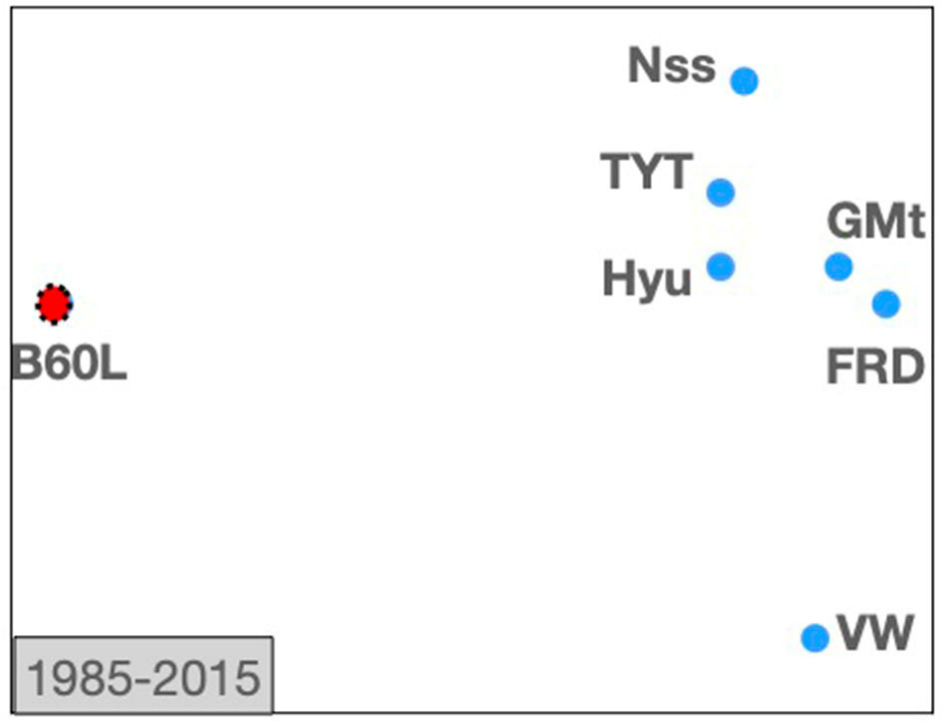
*TechMap*s from 1985 to 2015 for *B60L* IPC sub-class and the motor companies: *Ford, General Motors, Hyundai Motors, Nissan, Toyota Motors*, and *Volkswagen*.

The *TechMap* shows *Volkswagen* out of the group formed by the rest of the companies, and additionally it seems that the more active companies are *Hyundai, Toyota*, and *Nissan* (see Figure 7).

**Figure 8:**
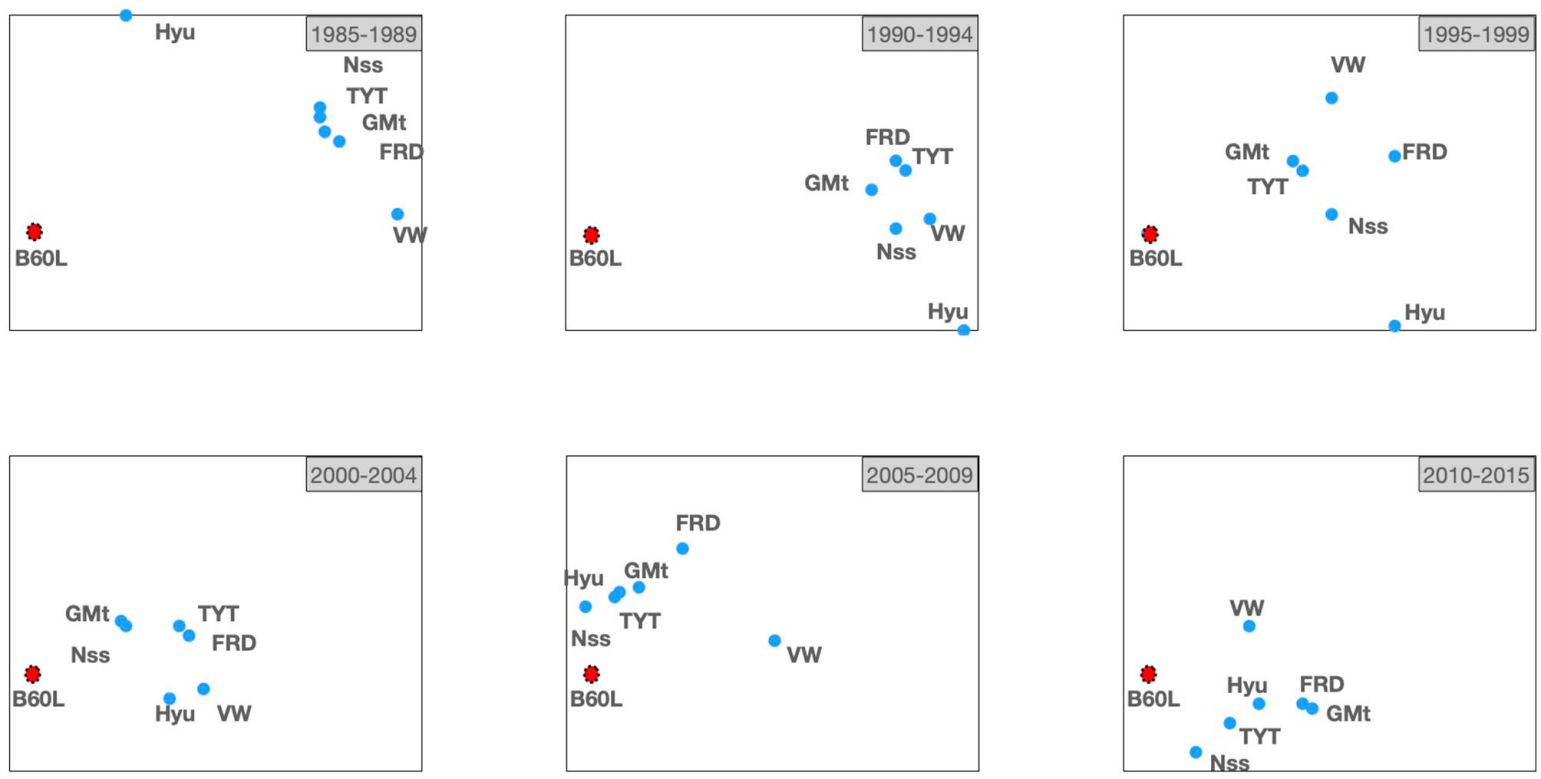
*TechMap*s evolution for 5-year intervals from 1985 to 2015 containing the *B60L* IPC sub-class and the motor companies: *Ford, General Motors, Hyundai Motors, Nissan, Toyota Motors*, and *Volkswagen*.

Now, we will visualize its evolution by computing a series of *TechMap*s with intervals of 5 years. Figure 8 shows (from right to left, and from top to bottom) the six *TechMap*s for 1985 to 1989, 1990 to 1994, 1995 to 1999, 2000 to 2004, 2005 to 2009, and 2010 to 2015.

It can be observed that, it is in the late 90s when some car companies started to approximate the *B60L* subclass. It appears that *Hyundai Motors* is far from this field until the beginning of 2000, when it joined the group of companies more active in electric vehicle technology. It also seems clear that *Nissan* stays from the late 90s as one of the companies more active in the field. It is also interesting to note how *Volkswagen* remains as one of the companies less active in this field until the 2010s.

We will now use the *F-TechMap* to *focus* on *B60L* for improving our perception of tech companies around electric vehicle technologies. As mentioned above^22^, the visualized items, in this case the motor car companies, will be located at actual distances from the *focus*, the electric vehicle technology *B60L*, and each item will be placed on the radius corresponding to most assigned IPC codes to it.

**Figure 9:**
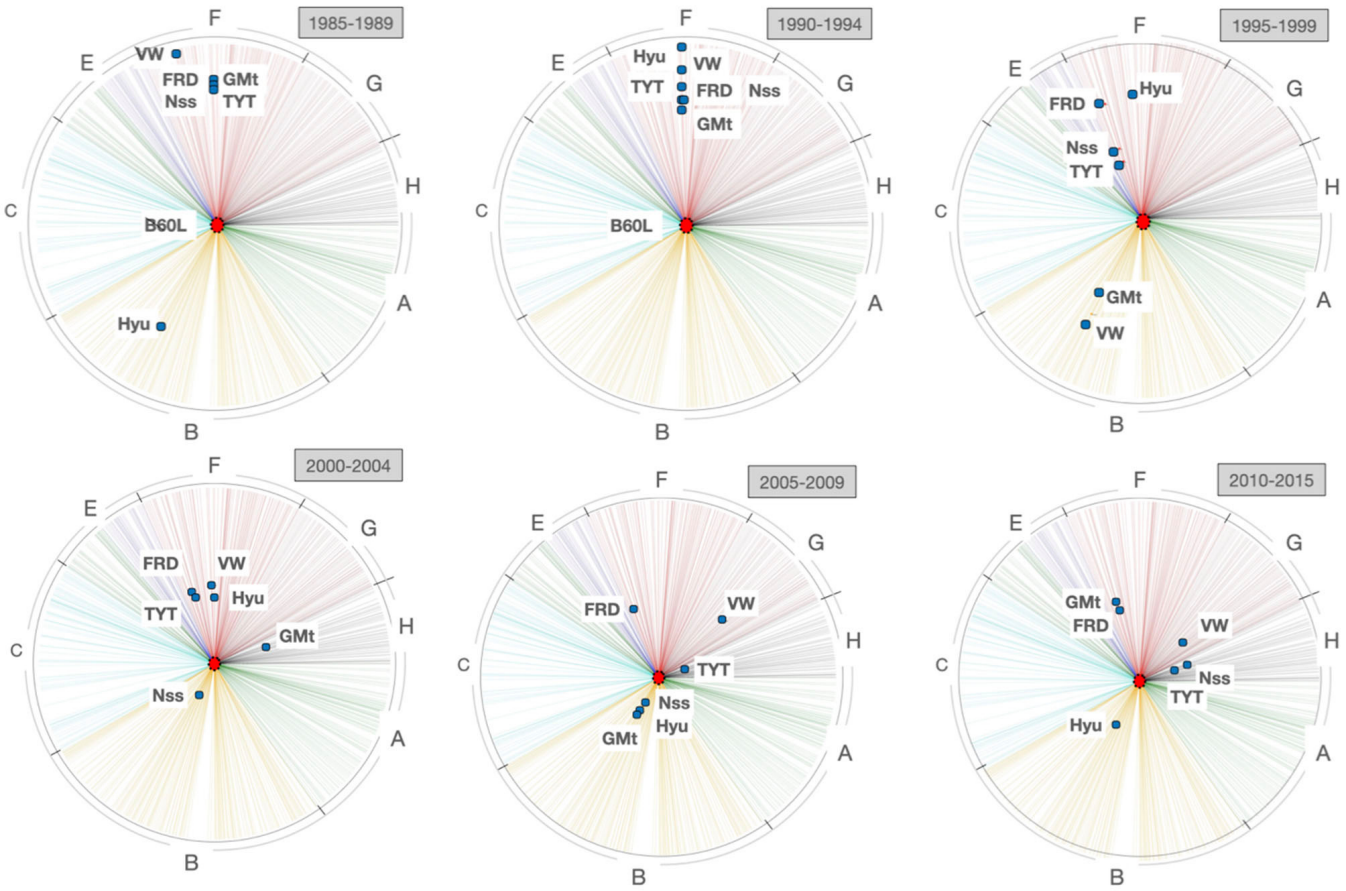
*Focused TechMap*s with focus in *B60L*. Evolution from 1985 to 2015 at 5-year intervals.

The *Focused TechMap*s confirm that from the interval 1995–1999, the car companies started to be more and more active in the field, pointing out *Toyota* and *Nissan*, and from the interval 2000–2005, also *Hyundai* (see in Figure 9 top right and bottom left graphs).

An interesting fact shown in these graphs is the change of the most important IPC section for some companies. In Figure 9, the three top graphs (1985–1999) place the companies on radii corresponding to sections B and F, which correspond to transporting and mechanical engineering technologies, respectively. These technical fields are traditionally considered as car technologies, whereas from 2000 (see in Figure 9 the three bottom graphs), some of these companies started to be located on electricity-physics technologies.

Note that by the interval 2010–2015, three companies are located in sections H or section G. In fact, *Nissan* and *Toyota Motors* are both located in section H (electricity), specifically in the subclass assigned to battery technology—*H01M*—. On the other hand, *Volkswagen* is located in section G (physics), specifically in the subclass assigned to digital data processing—*G06F*—. Somehow, these car companies are emerging as electrical technology developers in addition to, or above, mechanical technology.

Our maps from this study case align consistently with indicative events and data. For example, Figure 8 shows that our set of motor car companies started to approach B60L in the late 90s. Note some important industrial events for the automotive technologies in the late 90s such as, in 1996 *General Motors* produced the GM's *EV1*—first mass-produced electric vehicle—, and *Nissan* brought to the market the *Prairie Joy EV*—the first lithium-ion battery-powered car—, in 1997 the *Toyota*'s first *Prius* was marketed. Moreover, at the level of real data from patent portfolios, the focus on battery technologies and electronics from 2000 by our set of companies was above mentioned above.

## 4. Discussion

The visualization of proximity between technical fields and tech companies with respect to each other furnishes an insight into industrial and research developments. Such visualization provides information about firms' know-how, which facilitates potential collaborations and, technology opportunities and weakness. From this perspective, our work presents two complementary graphical tools for studying the relationship of technical fields and tech companies, and its evolution.

The information provided by our tool can help technology decision makers to understand competitors. For example, the evolution of motor car companies toward electricity technology can be explored and better understood by generating the *TechMaps* and then focusing on specific IPC codes or companies. Such graphs could also help to perceive the kind of knowledge that a specific technology gathers at a certain moment. Furthermore, the identification of these trends can be of great interest to researchers in the area of the history of technology.

The relative positioning of tech companies can be used to discover know-how or to explore complementarities between companies for potential mergers and acquisitions, or research collaborations. See, for example, the position of *Apple* in the *TechMap* and the *focused TechMap* of Figure 2 as a telephone set developer together with *Ericsson* in relation to *IBM* and *Microsoft*, two computing companies in principle similar to *Apple*.

It is important to highlight that our approach is to consider technical fields and companies (both defined by sets of IPC codes) as similar bodies, and they are represented by the same data structure, the *TechSpectrum*, namely the histogram of the IPC codes assigned to a set of patents and its citations mixed in a specific proportion, and ordered according to the IPC. This is a major difference with respect to the previous studies on visualization of technical fields and companies.

This conception of technical fields and tech companies as similar bodies of technology bring important implications in to the processing and visualization of technology. First, the processing is the same for technical fields and companies, so changes in time in both are tacking taken equally into account providing an overview on technical change as a whole. Second, observations for technical fields can be extended to companies.

Our *TechMap* has some distinctive features distinctive from traditional technology maps or landscapes. In *TechMap* the items, technical fields or companies, are located relative to each other, there is not absolute positioning. In previous maps or landscapes, the location of technology items is was computed and coordinate data are were assigned to it, so that if you added or removed an item, it is was added or removed to the map but the rest of it remained unchanged; whereas in *TechMap*s, removing or adding a technology item results in a computation of the new position of each item. Another distinctive feature is the capability to visualize tech companies in relation to technical fields at different levels of conceptual resolution by going deeper in the IPC classification levels. This operation is a sort of conceptual zoom rather than a zoom-in graphical operation as is usually the case in -known technology maps or landscapes such us the multiresolution zooming of Boyack et al. (2000).

The presence of clusters of technical fields within our *TechMap*s can show up the existence of some commonalities which can be useful for researchers and innovation managers to better understand these technical fields. See for example in Figure 4 how the IPC *subclass A61P, A61k*, and *A61Q* appear grouped. Or the, not obvious proximity between measuring systems for diagnosis—*A61B5*—and instruments for auscultation—*A61B7*—shown in Figure 5. It is interesting to highlight the exploration possibilities opened by visualizing with our two graphical tools tech companies in relation to technical fields, such as in Figure 2 where *IBM* and *Microsoft* appear grouped when they are positioned within a set of companies and the technical field of telephone sets—*H04M1*—.

## 5. Summary and future work

In this work we introduced a new graphical tool based on patent classification information to study technical fields represented by an IPC code (or a combination of codes) and tech companies. Each item, technical field, or company is characterized by a weighted mix of the classified (or assigned) patents and its cited prior art.

The first graph, the technology map—*TechMap*—, visualizes a set of given technologies, technical fields, and tech companies, positioning them in relation to each other. Our second graph, the focused TechMap, generates a visualization of the given technologies in relation to a selected one, the focus, which is located at the center of a circle with drawn radii corresponding to every IPC code. In this graph, the items are positioned at the computed distances from the focus, and on the radius representing its IPC code with the highest figures.

Although we have illustrated our tool with two case studies, namely medical and automotive technology, our tool has also been tested in a variety of fields such as heartbeat monitoring, 3D printing, hybrid vehicles, electronic devices, computer graphics, and or telecommunications to test for the ecological validity of our tool. So far, the authors found that this is a representative view in terms of ecological validity, except for the chemistry field which needs further dedicated testing due to its construct nature of how patents are triaged and presented. Concerning tech companies, we have tested the tool with numerous firms, and we have found that their use in our tool is constrained to firms active in patenting.

In the field of history of technology, our graphical tool contributes by facilitating the study of the evolution of specific technical fields, and to trace the divergence or convergence of tech companies. The contribution of the present paper lies in the creation of two new and complementary graphs based on patent classification information. The relative positioning of technologies (technical fields or tech companies) helps to better identify those that have some techniques in common because they appear close in the graphs, as well as to improve the understanding of the technical developments or trends in firms as was illustrated for motor car companies moving from pure mechanics to electrical technologies.

At present we are developing some algorithms for automatic clustering of items within *TechMaps*. Moreover, we foresee doing predictive modeling of the dynamics of the technologies to anticipate its their evolution, especially for tech companies.

Further research will be oriented to compute *TechMaps* with smaller time intervals in order to have more time resolution in the evolution perception to present animated versions of the visualizations and to investigate the modeling of trends.

In our study cases, we have highlighted some real data and events to give an indication of the consistency of our visualization tools. Further research will be carry carried out to systematically evaluate whether the company's perception of their technological developments corroborates with our tools.

We will explore the improvement of the similarity matrix using text similarity and citation network analysis of the patents classified in the respective IPC codes.

## Data availability statement

The datasets presented in this study can be found online via the following link: https://data.epo.org/expert-services/index.html.

## Author contributions

EP-M conceived the original idea and the presented method and visualizations. FL supervised the findings of this work, discussed the results, and worked on the manuscript. Processed the experimental data, performed the analysis, and wrote the paper with input from both authors. Both authors contributed to the article and approved the submitted version.

## Footnotes

^1^Bins are also known in other disciplines as intervals, classes, or buckets.

^2^Although the IPC has five levels—*Section, Class, SubClass, Group* and *SubGroup*—in the present work we have limited the analysis to the first four levels because the granularity at *SubGroup* level is too high and sensitive to the interpretation of the code definition.

^3^These are the number of IPC codes at each level on June 2023.

^4^ https://www.epo.org/searching-for-patents/business/patstat.html

^5^PatStat contains bibliographical data relating to more than 100 million patent documents from leading industrialized and developing countries. It also includes the legal event data from more than 40 patent authorities contained in the EPO worldwide legal event data.

^6^Our procedure and PatStat allows different time intervals.

^7^United States Patent and Trademark Office.

^8^For example A61C is closer to A61B, A61D, or A61L than to B61B, or A61B3 is closer to A61B5 than to A61C5.

^9^PatStat allows bigger time intervals than 1985–2015 and virtually any patent office.

^10^Section, Class, SubClass, and Group.

^11^The rationale behind this operation is to catch the importance of a group of IPC codes at a level when at a sub-level the group of codes is populated but very distributed—flattened—. If we only compute and display at a specific IPC level the importance of the group of IPC codes could be minimized by this flattened effect.

^12^ https://scikit-learn.org/stable/

^13^Appendix A contains the titles of all the IPC codes referenced in this paper.

^14^In brackets are the shorthands used in the graphs for each company.

^15^*A61B* has the following IPC *groups*: *A61B1, A61B3, A61B3, A61B6, A61B7, A61B8, A61B9, A61B10, A61B13, A61B16, A61B17, A61B18, A61B18, A61B34, A61B42, A61B46, A61B50*, and *A61B90*.

^16^G01: Measuring and testing technologies.

^17^A61K: Preparations for medical, dental, or toiletry purposes.

^18^A61P: Specific therapeutic activity of chemical compounds or medicinal preparations.

^19^A61Q: Specific use of cosmetics or similar toiletry preparations.

^20^*A61B5*: Measurement for diagnostic purposes.

^21^*A61B1*: Instruments for performing medical examinations of the interior of cavities or tubes of the body by visual or photographic inspection.

^22^See step 3 of *Focused TechMap* methodology on page 8.

## Appendix

Appendix A: Exact titles of cited IPC classification codes.

**A61** : MEDICAL OR VETERINARY SCIENCE; HYGIENE

**A61B** : DIAGNOSIS; SURGERY; IDENTIFICATION

**A61C** : DENTISTRY; APPARATUS OR METHODS FOR ORAL OR DENTAL HYGIENE

**A61D** : VETERINARY INSTRUMENTS, IMPLEMENTS, TOOLS, OR METHODS

**A61F** : FILTERS IMPLANTABLE INTO BLOOD VESSELS; PROSTHESES; DEVICES PROVIDING PATENCY TO, OR PREVENTING COLLAPSING OF, TUBULAR STRUCTURES OF THE BODY

**A61G** : TRANSPORT, PERSONAL CONVEYANCES, OR ACCOMMODATION SPECIALLY ADAPTED FOR PATIENTS OR DISABLED PERSONS

**A61H** : PHYSICAL THERAPY APPARATUS

**A61J** : CONTAINERS SPECIALLY ADAPTED FOR MEDICAL OR PHARMACEUTICAL PURPOSES; DEVICES OR METHODS SPECIALLY ADAPTED FOR BRINGING PHARMACEUTICAL PRODUCTS INTO PARTICULAR PHYSICAL OR ADMINISTERING FORMS; DEVICES FOR ADMINISTERING FOOD OR MEDICINES ORALLY; BABY COMFORTERS; DEVICES FOR RECEIVING SPITTLE

**A61K** : PREPARATIONS FOR MEDICAL, DENTAL, OR TOILET PURPOSES

**A61L** : METHODS OR APPARATUS FOR STERILIZING MATERIALS OR OBJECTS IN GENERAL; DISINFECTION, STERILIZATION, OR DEODORIZATION OF AIR; CHEMICAL ASPECTS OF BANDAGES, DRESSINGS, ABSORBENT PADS, OR SURGICAL ARTICLES; MATERIALS FOR BANDAGES, DRESSINGS, ABSORBENT PADS, OR SURGICAL ARTICLES

**A61M** : DEVICES FOR INTRODUCING MEDIA INTO, OR ONTO, THE BODY

**A61N** : ELECTROTHERAPY; MAGNETOTHERAPY; RADIATION THERAPY; ULTRASOUND THERAPY

**A61P** : SPECIFIC THERAPEUTIC ACTIVITY OF CHEMICAL COMPOUNDS OR MEDICINAL PREPARATIONS

**A61Q** : SPECIFIC USE OF COSMETICS OR SIMILAR TOILET PREPARATIONS

**B60** : VEHICLES IN GENERAL

**B60L** : PROPULSION OF ELECTRICALLY-PROPELLED VEHICLES; SUPPLYING ELECTRIC POWER FOR AUXILIARY EQUIPMENT OF ELECTRICALLY-PROPELLED VEHICLES; ELECTRODYNAMIC BRAKE SYSTEMS FOR VEHICLES IN GENERAL; MAGNETIC SUSPENSION OR LEVITATION FOR VEHICLES; MONITORING OPERATING VARIABLES OF ELECTRICALLY-PROPELLED VEHICLES; ELECTRIC SAFETY DEVICES FOR ELECTRICALLY-PROPELLED VEHICLES

**G06** : COMPUTING; CALCULATING OR COUNTING

**H01** : BASIC ELECTRIC ELEMENTS

**H01M** : PROCESSES OR MEANS, e.g., BATTERIES, FOR THE DIRECT CONVERSION OF CHEMICAL ENERGY INTO ELECTRICAL ENERGY

**H04** : ELECTRIC COMMUNICATION TECHNIQUE

**H04M** : TELEPHONIC COMMUNICATION

**H04M1** : SUBSTANTION EQUIPMENT, e.g., for use by SUBSCRIBERS
